# A Case in Which the Skin of a Child Born of White Parents Was so Discolored as to Suggest the Idea of Mixed Blood

**Published:** 1845-06

**Authors:** John Stackhouse

**Affiliations:** Patriot, Indiana


Art. III.—*A Case in which the Skin of a Child born of white parents was so discolored as to suggest the idea of mixed blood.* By John Stackhouse, M.D., of Patriot, Indiana.

The case, the particulars of which I am about to relate, possesses interest on account of its relations to medical jurisprudence, and is, therefore, probably worthy of being preserved.

A family moved into this neighborhood sometime since, and not long after they came, the mother, who was pregnant, was taken with a bilious affection which, though slight at first, finally confined her to her bed. One of the most distressing symptoms in her case was a difficulty of breathing, attended with something like spasms, when she was in the recumbent posture. This, I supposed, grew out of the pressure of the fœtus on the abdominal aorta. In about three weeks from the supervention of her indisposition she was delivered of a female child. At first, being occupied with the case of the mother, I paid but little attention to the infant, but on inspecting it closely the next day, I was induced by its appearance to suspect that it might be a mulatto. Its hair was very black, long, straight and fine, feeling somewhat woolly. The white of its eyes was clear, and showed more than it usually does in the white race. The iris and pupil had the dark complexion seen in the mulatto; the head, from the forehead to the occiput was longer, and from temple to temple narrower, than common; the base of the nose was well raised, and its point not broader or flatter than is usual in white children; its lips, if any difference, were somewhat thicker, and on the inside were of a bluish color; the hands and feet were delicate, and the latter corresponding in the heel and instep to the Caucasian form. The skin of the face was as dark as it is found on the mulatto, but there was something peculiar in its appearance, such as might be given by dusting the surface with a puff-ball. Along the back the color was very dark, as also down the belly; the remainder of the skin was of the mulatto color.

The meconium not having been discharged, I ordered castor oil, but my directions were not obeyed, and on the second day an evacuation of it took place, from the action of the colostrum. The child was observed to be unusually drowsy, to sleep more than was natural, and was roused by the mother, even to nurse, with difficulty. Something like spasms were early seen when she was raised up; at the same time her bowels were irregular, though the stools were not unnatural in color, nor was the urine discolored, nor her diapers stained by that secretion.

Symptoms of bad health continued for three weeks, when she was seized suddenly with an alarming convulsion. I found her shortly after laboring under what might have been taken for a case of spasmodic croup. An emetic of ipecacuan was administered, which operated twice, with the relief of the symptoms, for the time. In the evening of the same day, however, the spasm returned, the breathing becoming very difficult. I resorted to blood-letting and a warm bath, from which she obtained pretty speedy relief, and passed a comfortable night, under the influence of minute doses of calomel and ipecac. The following day, about 11 o'clock, she was seized with another convulsion, which continued, with occasional intermissions, until 11 o'clock at night, when it subsided; but at the same hour the next day the spasms again supervened, thus seeming to have assumed a periodical character, recurring at regular intervals. In this manner they continued to come on for four days, and were not followed by fever. After this time, they began to anticipate, appearing daily at an earlier hour, and extending farther into the night, and were succeeded by fever. On the tenth day after she was attacked by convulsions she died.

The report having become common that the child was a mulatto, the parents agreed, the day after burial, that the body should be disinterred and examined. Six physicians were present at the autopsy.

The first circumstance which struck us, was the staining of the shroud of a deep saffron color, by a fluid which had run out of the nose. On opening the trachea an unusual amount of yellow mucus was found lining its walls. The bloodvessels of the brain appeared to be in a state of congestion, but the substance of that organ exhibited a healthy appearance; a tea-spoonful of water was contained in the right ventricle.

On laying open the abdominal cavity, the bowels presented a delicate tinge of yellow; the liver was cut into and appeared harder than natural, and otherwise unhealthy. The examination was not carried further.

Could this have been a case of jaundice, unaccompanied by the usual discoloration of the eyes, and of the urine?

Is it not possible that the bilious disorder of the mother was communicated to the fœtus in utero, which came thus to present the appearances described?

Are there any certain marks for distinguishing a mulatto from a white child after several days' interment? Is the hair of mulattoes black, straight, and generally longer than the hair of white children; or is it curled at birth; or does it become curly in three or four weeks? Have they more black upon the back and abdomen than on other parts, and do their feet partake of the peculiarities of the Caucasian foot?

These are some of the questions which seem naturally to arise on a review of this case, and concerning which it would be well if the profession had more precise information, coming up as they occasionally do in courts of justice, and eliciting from medical witnesses the greatest contrariety of opinion. The reader will perhaps decide at once, that all the appearances exhibited by this infant arose from disease, and this decision may be a correct one; but it is enough to show that the question is not of such easy solution, that the persons who inspected the body of the child did not come unanimously to that conclusion.

